# Annotations

**Published:** 1892-05-14

**Authors:** 


### Annotations.

**Male and Female Brains.**

The unscientific emotions educed in certain quarters by the publication of Sir James Crichton-Browne's lecture on "Sex in Education," having now subsided to a certain extent, it will be interesting to consider a few of the actual facts ascertained and set forth by the lecturer. There can be no doubt, for instance, that in the matter of weight there is a marked difference between the male and the female brains. The difference corresponds to a difference in size. The female brain, in human creatures, is smaller than that of the male, and it is also lighter. The difference in weight is considerable. The average man's brain is between four and five ounces heavier than the average woman's. The reason, it may be said, is that the woman herself is smaller than the man in size and weight. That accounts partly for the difference, but not entirely. It is shown by many and careful observations that if women were as tall as men, and as heavy, the average weight of their brains would still be smaller than that of men by more than an ounce. The diminished size and weight of the brain is said to be a fundamental sexual distinction in the human species. It is not peculiar to civilised men and women, but is found universally among savages, wherever sufficient observations have been made. The difference in weight does not exhaust the catalogue of diversities. There is said to be also a difference of balance between the various parts of the compared brains. The occipital lobes, which preside chiefly over the physical functions of the organism, are declared to be more voluminous in the female than in the male; a physiological fact which is contrary to common belief. A third striking diversity is that whilst the white matter of the brain, which has no thought function, is almost identical in weight in the two sexes, the specific gravity of the grey, or thought matter, is decidedly higher in the male than in the female. Now, these are facts. It is true that Sir James Crichton Browne has set them forth, but it is not true that he has originated them. If any lady is disposed for a quarrel on the occasion she should not quarrel with Sir James Crichton Browne, but with niggard nature, or with Mr. Mathew Arnold's unchivalrous "stream of tendency." It appears to be unquestionable that in purely intellectual endowment the man is superior to the woman. On the other hand, in the equally noble emotional capacity the woman is superior to the man. If these be the facts, as they certainly appear to be, it is well that both the sexes should recognise and make the best they can of them.

**Brain Blood in Men and Women.**

He was a very ancient physiologist who first declared "The blood is the life." It is true in a very fundamental sense that the blood is the life of the brain as well as of the body generally. Sir James Crichton Browne and Dr. Sidney Martin have spent three or four years in trying to ascertain the relative capacity of the chief blood vessels that supply the brain in men and women. They have probably been surprised to find that whilst the absolute capacity of these arteries is greater in men, the vessels are really more capacious in women in proportion to the weight of brain that is to be supplied. That is to say, nature has provided women with a larger brain blood supply than that which she has given to men. On the other hand, the quality of female blood is poorer than that of the male. It is extremely interesting, too, to note another set of facts in this connection. The blood vessels which supply the cerebral or thinking part of the male brain are both absolutely and relatively much larger than those which supply the thinking part of the female brain. But, by way of set-off, the occipital or emotional and sensory lobes in the female brain are much more bountifully supplied with blood than the corresponding parts in the male. Of course there is a good deal of what physiologists call "anastomosing" in the vessels of the brain, and, consequently, a considerable degree of mixing of the blood. It is still the fact, however, that nature in her discretion has provided more nourishment for the emotional and sensory part of woman's brain than for the more purely thinking part, and conversely that she has made the larger provision for the thinking part of man. It is not without physiological warrant, therefore, that we speak of the "dry light of reason" in men. These facts have a fundamental quality about them. They are full of the deepest possible significance for those who have eyes to see. What Nature herself has established the cleverest of men and women will find it difficult to disestablish. It must still be the business of the woman to make the domestic hearth radiant with her love; the man must fare forth to sterner conflict. So Nature has willed it.

**The "Daily Chronicle" & the Scotch Miners.**

The *Daily Chronicle* expresses both surprise and incredulity at the statements made in the Scottish Sketch, entitled "Wages v. Comfort," which appeared in last week's The Hospital. The incredulity is so natural that the writer of the article—who would have been quite as unbelieving three years ago—cannot resent it, and answers only by the invitation "Come and see." The facts are easily verifiable. The writer wonders that there is not a rush of the unemployed to Lanarkshire. As a matter of fact, a considerable number of Irishmen join the ranks of the Scotch colliers every year; English immigrants are more rare, but when they come they usually bring a higher standard of cleanliness and comfort with them. At some pits, however, the men propose to inflict a fine of a guinea (to be drunk in good fellowship) on every man who is not the son of a collier, on his going down the pits for the first time. A tax like this will be prohibitive to most aspirants. It might be supposed that masters and managers would prevent such injustice. But when prices are high the masters are too anxious to get their wares into the market to quarrel readily with the men who dig out the coal. The "diplomacy" by which a boy can earn a man's wage, and which puzzles the leader writer of the *Chronicle* is simple enough. The father, having finished the amount of work he is allowed to put out in a day, supplements his son's work and brings it up to an equal quantity. The boy is then rated as a man, and receives a man's wage, which practically goes in to the father's pocket. This would not affect the average wage as stated in the Board of Trade returns, though it would add materially to the adult miner's income. The system of sub-contract is practised in many large works. By it the sub-contractor, who is of the mining class himself, and knows the nature of his fellows, makes very considerable sums, but except in the physical sense he does not "sweat" his men. These will not work steadily for a fixed wage, but can be bribed to enormous exertions by the promise of a gratuity, which is never entered on the balance-sheet as wages. Nor are statistics always thoroughly accurate. King Gama's sneer at "the Income-tax returns" is justified by common experience. A miner certainly would not pay income-tax; he would sooner "flit" to another colliery every year. The *Chronicle* advises the Lanarkshire miners to join the Miners' Federation. This might be to their benefit morally; but materially—how? They are in no "pitiable plight" according to their own ideas. They have enough to eat and drink, and they want little more. They are content with their lot. To others it seems miserable and degraded, and steady efforts are being made to raise them from it.

